# A prospective double-blinded randomized control trial comparing erector spinae plane block to thoracic epidural analgesia for postoperative pain in video-assisted thoracic surgery

**DOI:** 10.15537/smj.2023.44.2.20220644

**Published:** 2023-02

**Authors:** Jeong-Min Hong, Eunsoo Kim, Soeun Jeon, Dowon Lee, Jiseok Baik, Ah-Reum Cho, Jeong Su Cho, Hyo Yeong Ahn

**Affiliations:** *From the Department of Anesthesia and Pain Medicine (Hong, Kim, Jeon, Lee, Baik, A. R. Cho), School of Medicine, Pusan National University; from the Department of Anesthesia and Pain Medicine (Hong, Kim), Biomedical Research Institute, Pusan National University Hospital; from the Department of Thoracic and Cardiovascular Surgery (J. S. Cho, Ahn), Pusan National University Hospital, Busan; from the Department of Anesthesia and Pain Medicine (Jeon), School of Dentistry, Kyungpook National University, Daegu, Korea.*

**Keywords:** epidural analgesia, erector spinae plane block, patient-controlled analgesia, morphine, multimodal analgesia, pain, postoperative, programmed intermittent bolus infusion, thoracic surgery, video-assisted

## Abstract

**Objectives:**

To compare the analgesic efficacies of erector spinae plane (ESP) block and thoracic epidural analgesia (TEA) in video-assisted thoracic surgery (VATS).

**Methods:**

Sixty patients undergoing VATS received patient-controlled TEA with a basal rate of 3 ml/hour (h), a bolus of 3 ml (Group E), or ESP block with programmed intermittent bolus infusions of 15 mL/3 h and a bolus of 5 ml (Group ES) for 2 postoperative days. The primary outcome was to compare pain scores at rest 24 h postoperatively between the 2 groups. Secondary outcomes included NRS score for 48 h, procedural time, dermatomal spread, use of rescue medication, adverse events, and patient satisfaction.

**Results:**

Patients with continuous ESP block had a higher NRS score than those with TEA but no statistical difference at a specific time. The dermatomal spread was more extensive in the TEA group than in the ESP block group (*p*=0.016); cumulative morphine consumption was higher in the ESP block group (*p*=0.047). The incidence of overall adverse events in the TEA group was higher than in the ESP block group (*p*=0.045).

**Conclusion:**

Erector spinae plane block may be inferior to TEA for analgesia following VATS, but it could have tolerable analgesia and a better side effect profile than TEA. Therefore, it could be an alternative to TEA as a component of multimodal analgesia.

**L**ung resection surgery causes considerable postoperative pain, which can interfere with deep breathing or coughing during the postoperative period, and it can lead to postoperative pulmonary problems, such as atelectasis, lung infection, and respiratory failure.^1^ Although video-assisted thoracic surgery (VATS) is typically regarded as less invasive and painful, moderate-to-severe postoperative pain is still to be expected.^2^ Recently, appropriate and efficient postoperative pain management has become a requirement for improved recovery after surgery in thoracic surgery.^3^ It can improve surgical outcomes, reduce complications, and reduce hospital stays. Thus, inadequate pain relief following VATS exposes the patients to postoperative morbidities and affects the quality of the patient’s recovery.

Thoracic epidural analgesia (TEA) and thoracic paravertebral blocks (TPVB) have been used widely to manage postoperative pain in thoracic surgery. However, these gold-standard analgesic techniques are often challenging to perform and may induce complications, including dural puncture, nerve injury, epidural hematoma, infection, hypotension for TEA or pneumothorax, local anesthetic toxicity, inadvertent spinal or epidural block for TPVB.^4^ Thus, various regional nerve blocks, including intercostal nerve block, serratus plane block, pectoral nerve block, and retrolaminar block can be performed as alternative analgesic methods.^5^ The erector spinae plane (ESP) block is a recently described interfascial procedure where local anesthetics (LA) are injected between the erector spinae muscle and the tip of the transverse process (TP).^6^ This block is simple to perform, and numerous studies have established the analgesic efficacy of ESP block in a variety of therapeutic settings.^6-8^ However, because most earlier published studies were case reports,they could not provide a clinical basis for the effectiveness and safety of ESP blocks.^9^ Therefore, we performed a comparative study to evaluate the analgesic efficacy between continuous ESP blocks and TEA in patients with lung cancer receiving VATS.

## Methods

This prospective, randomized, double-blind study was approved by Pusan National University Hospital’s Institutional Review Board (No. H-1807-029-069) and was registered with cris.nih.go.kr (registration number: KCT0003836; date of registration: August 31, 2018). This study was carried out in accordance with the principles of the Helsinki Declaration. An informed consent was obtained from 60 patients with the American Society of Anesthesiologists physical status classification I-II aged 18 to 85 years and scheduled for lung resection using VATS from October 2018 to May 2019 in Pusan National University Hospital, Busan, Korea. Patients who met any of the following criteria were excluded: inability to understand or give informed consent; chronic use of opioids or steroids; the presence of heart, liver, or kidney function abnormalities; infection at the site of the analgesic procedure; abnormal coagulation profile; or a body mass index higher than 30 kg/m^2^.

Two groups of patients having VATS were randomly assigned: Group E (n=30) got continuous epidural analgesia, while Group ES (n=30) received continuous ESP block. They were allocated to each group using block randomization tables generated using Randomization.com (http://www.randomization.com). A single anesthesiologist performed all analgesic procedures before inducing general anesthesia in the operating theatre. Ultrasound was used to identify vertebral levels in both groups and guide needle advance and catheter placement in the ESP block group. Fluoroscopy was applied to check the catheter tip position in both groups and confirm the epidural space in the epidural group. Ultrasound and fluoroscopy were used with all patients, so patients could not tell which procedure was performed. Researchers who did not attend the analgesic procedures recorded the postoperative pain score and complications. In addition, patient-controlled analgesia (PCA) pump settings and drugs were also recorded using an electronic data collection tool.

### Analgesic procedure

#### Group E

In a lateral decubitus position, the interlaminar space between the 6th and 7th thoracic vertebrae was identified using ultrasound by counting ribs and TPs of thoracic vertebrae downward from the first rib. The patient underwent skin disinfection and received a block, and an 18G Tuohy needle (Perifix^®^ Soft Tip 700 Filter Set; B. Braun, Melsungen, Germany) was inserted via a paramedian approach. Using the loss-of-resistance approach, the epidural space was detected, and a multi-orifice catheter was placed around 3 cm beyond the needle’s tip. After measuring the location of the catheter tip using fluoroscopy with a contrast agent, a test dose of 3 mL 2% lidocaine with 15 µg epinephrine was administered to ensure that the catheter was not in the subarachnoid space or epidural vein. A loading dose of 6 mL 0.375% ropivacaine was administered 30 minutes before the end of surgery. A PCA system was used to deliver 0.2% ropivacaine (Accumate 1200; Woo Young Medical, Seoul, Korea; 250 mL 0.2% ropivacaine, background infusion rate 3 mL/hour (h), bolus volume 3 mL, lock-out interval 20 minutes) for 2 postoperative days.

#### Group ES

In a prone position, the TP of the 6th thoracic vertebra was identified using ultrasound with downward counting from the 1st rib. Following standard skin disinfection, a linear transducer (6-13 MHz HFL38x; Fujifilm SonoSite, Bothell, WA) was placed parallel to the thoracic spine to visualize the 6th TP, and an 18G Tuohy needle was inserted by the in-plane technique, in a caudad to cephalad direction. After the needle tip touched the 6th TP, 10 mL of normal saline was applied to confirm spreading to the deep part of the erector spinae muscle and to make space for catheter insertion. Then, a multi-orifice catheter was implanted 3 cm beyond the needle tip. The anesthesiologist checked the location of the catheter tip by fluoroscopy. A loading dose of 20 mL 0.375% ropivacaine was injected 30 minutes before the end of surgery. A programmed intermittent bolus infusion (PIBI) of 15 mL 0.2% ropivacaine every 3 h was started 3 h after injecting the loading dose (20 mL) using a PCA pump with a 5 mL bolus and a lock-out interval of 20 minutes for 2 postoperative days.

#### General anesthesia and surgery

General anesthesia was induced using a target-controlled infusion (TCI) pump with effect site concentration of 3-4 µg/mL for propofol and 3 ng/mL for remifentanil, with 0.6 mg/kg rocuronium. Maintenance of anesthesia was accomplished by titrating propofol by TCI according to a bispectral index response of 40 to 60. Depending on the strength of the surgical stimulus, the effect-site TCI of remifentanil was titrated. All patients took 0.3 mg ramosetron to prevent postoperative nausea and vomiting before extubation.

Surgeons performed VATS using 3 ports located at 4, 6, and 7 intercostal space; a 40-100 mm length working window and 2 small ports for thoracoscopy and instrument. A chest tube was inserted via one of the port incisions at the conclusion of the operation.

Following surgery, patients were transported to the post-anesthesia care unit or critical care unit. Both groups were given 1 g intravenous propacetamol hydrochloride (Denogan) 3 times per day. Patients were treated with 3 mg morphine sulfate as a rescue analgesic medication when they still had NRS score of 4 or more 20 minutes after a patient-administered bolus of PCA system.

The primary outcome measure in this study was comparing the numerical rating scale (NRS) scores at rest 24 hours postoperatively between the 2 groups. The NRS score was measured with a 0-10, visually enlarged, laminated numerical rating scale for minimizing the effect of the patient’s awareness at 1, 3, 6, 12, 24, and 48 hours postoperatively. Secondary outcomes were divided into 4 categories: i) block characteristics, ii) rescue medication, iii) adverse events, and iv) patient satisfaction.

In block characteristics, the procedure time (from skin block to catheter indwelling) and the location of the catheter tip were recorded. The dermatomal sensory range after loading dose administration was measured using the pinprick test 1 hour after surgery. Data on rescue analgesic medication were taken from the electronic medical record (EMR) and nurses during the follow-up period. Information on adverse events was collected from patients, nurses, and EMR. Nausea was defined when rescue antiemetics were administered. Hypotension was diagnosed when systolic blood pressure was less than 90 or vasopressor was needed. Patient satisfaction was checked with a 5-point scale (5=very satisfied, 4=satisfied, 3=neutral, 2=unsatisfied, 1=very unsatisfied) just after the procedure and at last follow-up. Postoperative follow-up visits were done 1, 3, 6, 12, 24, and 48 hours after surgery.

#### Statistical analysis

The G * power (version 3.1, University of Heinrich-Heine, Dusseldorf, Germany) was used to estimate sample size based on the results of the previous study.^10^ According to the reference research, the expected NRS at 24 h postoperatively was 3.08 ± 0.70 for Group E and 2.36 ± 1.07 for Group ES. With an α-value of 0.05 and a β-value of 0.2, 26 participants per group were required. Therefore, we decided on a sample size of 30 participants per group, assuming a 15% dropout rate.

All analyses were carried out using SPSS Statistics (version 22; IBM, Armonk, NY) and MedCalc^®^ (version 20; MedCalc Software Ltd, Ostend, Belgium) and were based on a per-protocol analysis method. Continuous variables were reported as median and first and third quartiles (Q1, Q3), mean and standard deviation (SD), or mean difference, and 95% confidence interval (95% CI). Categorical data were reported as absolute numbers with percentages. Following the normality test, nonparametric data were assessed using the Mann–Whitney U test, whereas gaussian-distributed data were assessed using an independent t-test. The Chi-squared test (the 2 × 2 contingency table: Yates’ continuity correction), Fisher’s exact test, or the Mantel-Haenszel trend test were used to evaluate categorical data. After aligned rank transformation, a 2-way repeated-measures analysis of variance (RM-ANOVA) was carried out for the NRS analysis to examine the interaction between time and group; at each measurement point, the Mann-Whitney U test and false discovery rate (FDR) correction was performed to compare the 2 groups. In addition, the non-inferiority test was performed for postoperative pain score differences. When the 95% CI’s upper limit was less than one, non-inferiority was concluded. Two-sided *p*-value of <0.05 were regarded as statistically significant.

## Results

**Figure 1:**
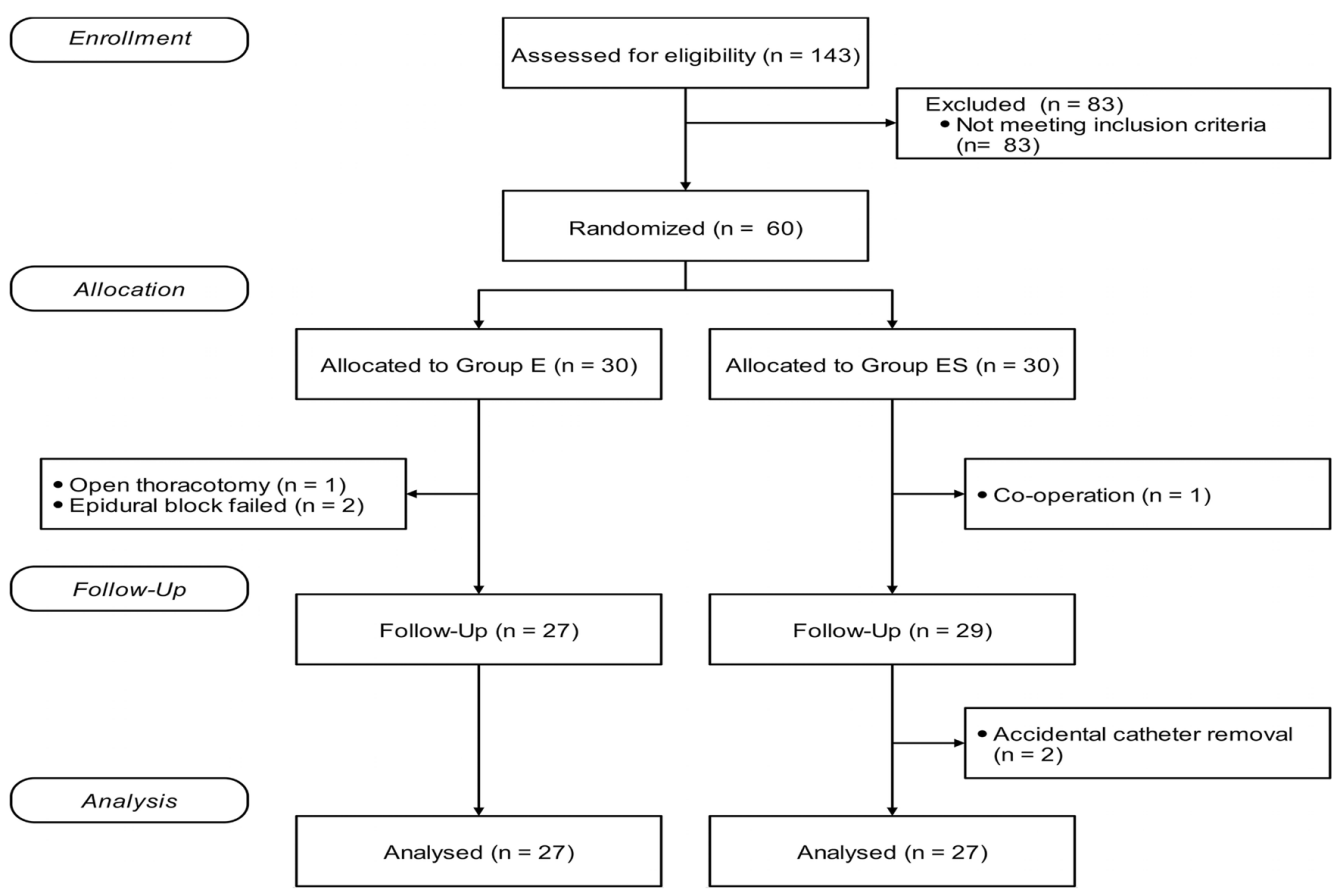
- The consolidated standard of reporting trial flow diagram of this study.

A total of 143 patients were assessed for inclusion in this study. Sixty patients were recruited, and 30 were assigned to each group at random. Three patients in the epidural group were excluded from the study due to failed epidural block and open thoracotomy. Three patients in the ESP block group were excluded due to thyroid co-operation and accidental catheter removal. Fifty-four subjects finally completed the study and were analyzed (Figure 1).

**Figure 2:**
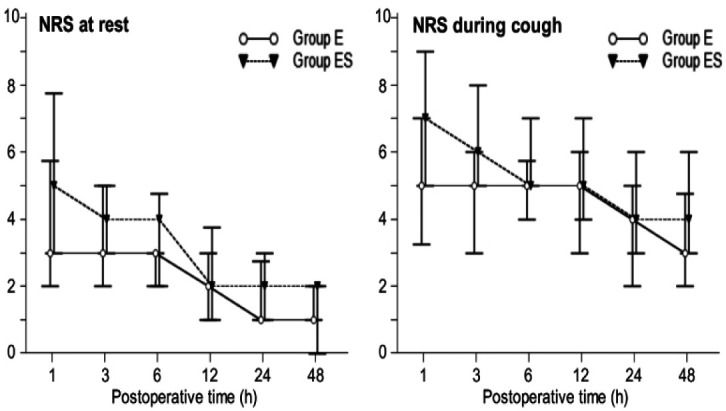
- Postoperative pain score at rest and during coughing for 48 hours (h). Values are expressed as median with the first quartile (Q1) and third quartile (Q3). Two-way repeated-measures analysis of variance (RMANOVA) after aligned rank transformation was performed to analyze the interaction between time and group. The Mann–Whitney U (MWU) test with false discovery rate (FDR) correction was used to compare the 2 groups at each measuring point. Group E; continuous thoracic epidural analgesia (TEA) group, Group ES; continuous erector spinae plane (ESP) block group, NRS; numerical rating scale. *p<0.05

**Figure 3:**
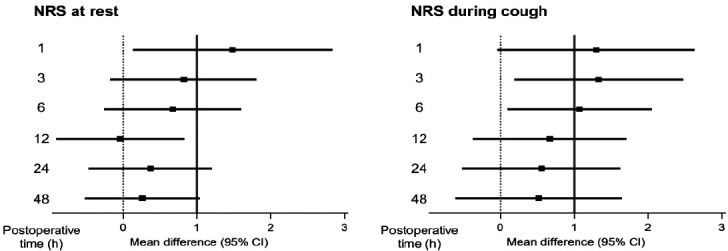
- Non-inferiority test of postoperative pain score at rest and during coughing for 48 hour. Values are expressed as mean difference (95% confidence interval [95% CI]). Non-inferiority was determined when the upper boundary of the 95% CI was <1. Group E; continuous thoracic epidural analgesia (TEA) group, Group ES; continuous erector spinae plane (ESP) block group, NRS; numerical rating scale.

The demographic characteristics and surgical data of the 2 groups were equivalent (Table 1). The NRS score at rest 24 h postoperatively had no statistical difference between groups. The NRS pain score at rest and while coughing showed differences over time (*p*<0.001 and *p*<0.001) and between Groups E and ES (*p*<0.002 and *p*<0.001). Although Group ES had a higher NRS score than Group E at rest, we could not find a statistical difference at a specific time (Figure 2). Further statistical analysis with the non-inferiority test, the upper limit of the 95% CI in mean difference in the pain at rest between 2 groups, except 12 hours, was higher than the pre specified non-inferiority margin (δ=1), non-inferiority was not established (Figure 3).

**Table 1:** - Demographic data and surgical characteristics of patients.

Demographics	Group E (n= 27)	Group ES (n=27)	*P*-value
Gender (M/F)	13 / 14	15 / 12	0.785b
ASA (I/II)	2 / 25	0 / 27	0.471b
Age, years	64.3 ± 10.3	66.5 ± 7.0	0.367
Height, cm	161.8 ± 9.9	161.7 ± 7.8	0.988
Weight, kg	64.5 ± 10.6	61.7 ± 8.6	0.288
BMI, kg/m2	24.6 ± 2.7	23.6 ± 2.6	0.177
***Surgical characteristics***			
Anesthesia time, min	208.3 ± 54.8	211.9 ± 63.9	0.829
Operation time, min	15261 ± 47.0	162.2 ± 59.5	0.512
Propofol infusion, mg	1415.0 (1185.0, 1661.0)	1394.0 (1294.0, 1651.0)	0.716a
Remifentanil, µg	726.8 (585.0, 1134.0)	901.0 (642.0, 1095.0)	0.341a
Rocuronium, mg	70.0 (60.0, 90.0)	75.0 (65.0, 90.0)	0.690a
Surgeon (A/B)	17 / 10	20 / 7	0.558b
***Type of operation***			0.706b
Lobectomy	19	18	
Segmentectomy	2	1	
Wedge resection	4	7	
Lobectomy + part of other lobes	2	1	

Normally distributed data were presented as mean ± standard deviation and analyzed using an independent t-test. Nonparametric data were presented as the median and interquartile range (Q1, Q3) analyzed using the Mann–Whitney U test (a). Categorical data were reported as absolute numbers and analyzed using the chi-square test (with Yates’ continuity correction for the 2 × 2 contingency table) or Fisher’s exact test (b).

The extent of the sensory dermatome was wider in Group E than in Group ES (*p*=0.016). Still, the 2 groups had no other significant differences in block characteristics (Table 2). Group ES showed significantly more cumulative morphine consumption than Group E at postoperative 48 hours (*p*=0.047, Table 3). The incidence of postoperative adverse events was higher in Group E compared with Group ES (*p*=0.045). Satisfaction regarding postoperative pain management was higher in Group E compared with Group ES *p*=0.023, Table 3).

**Table 2:** - Procedural time and dermatome spread of sensory block between groups.

Characteristics	Group E (n=27)	Group ES (n=27)	*P*-value
Procedural time (sec)	145.0 (105.0, 170.0)	130.0 (97.0, 140.0)	0.139^a^
***Tip of catheter***			1.000^b^
T4	10	9	
T5	13	14	
T6	3	4	
T7	1	0	
Dermatome range of sensory 1 hr after loading dose	5.0 (4.0, 6.0)	4.0 (4.0, 5.0)	0.016 ^a^

Continuous variables were presented as the median with interquartile range (Q1, Q3) and analyzed using the Mann–Whitney U test (a). Categorical data were reported as absolute numbers and analyzed using the Mantel–Haenszel trend‐test (b).

**Table 3:** - Use of analgesic drugs, adverse events, and patient satisfaction during the postoperative period.

Parameters	Group E (n = 27)	Group ES (n = 27)	*P*-value
Use of rescue drug (Yes/No)	19 / 8	22 / 5	0.524^b^
Time to first rescue drug injection, min	55.0 (0, 150.0)	25.0 (5.0, 90.0)	0.417^a^
Total morphine consumption (mg)	3.0 (0, 4.5)	6.0 (3.0, 9.0)	0.047^a^
***Incidence of adverse events***			
Total	15	6	0.045^a^
Nausea	7	5	0.852^b^
Dizziness	7	3	0.293^b^
Hypotension	2	0	0.471^b^
Arrhythmia	0	0	
Syncope	0	0	
***Satisfaction (5-point scale)***			
Procedural (1/2/3/4/5)	0/0/2/11/14	0/0/7/14/6	0.054^b^
Postoperative pain (1/2/3/4/5)	0/0/2/14/11	0/1/7/16/3	0.023^b^
Adverse effect (1/2/3/4/5)	0/2/3/8/14	0/3/3/12/9	0.569b

Continuous variables were presented as median with interquartile range (Q1, Q3) and analyzed using the Mann–Whitney U test (a). Categorical data were reported as absolute numbers and analyzed using the chi-square test or Fisher’s exact test (b). Min: minimum

## Discussion

In this study, we examined the effectiveness of ESP blocks and TEA as postoperative analgesics in patients undergoing VATS. Continuous ESP block provided inferior postoperative analgesia to TEA regarding analgesic efficacy in the immediate post-operative period following VATS, as shown by the NRS score at rest and during coughing for 48 hours postoperatively. Furthermore, ESP block needed more opioid consumption than the TEA group. However, the NRS score difference between groups was smaller than 2/10, and the TEA group had a greater incidence of overall adverse events than the ESP block group.

Forero et al^6^ first described ESP block for thoracic neuropathic pain. Erector spinae plane block is an emerging regional block in which LA is deposited in the interfascial plane between the erector spinae muscle and the transverse process of the vertebra. Erector spinae plane blocks have been utilized for acute pain control following surgery and chronic pain control in several body areas and for various surgeries, owing to their simplicity and safety in the last 2 years.^11^ In many articles published describing the application of ESP blocks for VATS, the procedure is performed with a single injection or continuous infusion method at the 5th thoracic vertebra TP, which provides multi-dermatomal spread and lower pain score, and decreased opioid consumption.^6,7,12-18^ In our study, the ESP block group described a reasonable analgesic efficacy, cephalocaudal spread covering an average of 4 dermatomes, and an improved side effect profile.

Previous research has suggested that the ESP block may function by blocking the dorsal and ventral rami of the thoracic spinal nerves as well as sympathetic nerve fibers.^6,19^ Cadaveric data show heavy dye staining anterior to the erector spinae muscles and intercostal muscles, as well as around the costotransverse junction, indicating spreading in the paravertebral space in anatomical dissection.^6^ Following this evidence, the recent cadaveric study described that an ESP block with 20 mL of injectate provided neural foraminal and epidural spread across 2 to 5 vertebral levels in magnetic resonance imaging.^20^ In contrast with this evidence, other cadaveric research demonstrated that 20 mL of dye infused between the erector spinae muscle and the transverse process resulted in extensive cephalocaudal and medial-to-lateral spread but no anterior spread to the paravertebral space.^21^ Thus, local anesthetics of ESP block may spread into the paravertebral area through porous ligamentous tissues. However, the extent of distribution into this space could be more limited than that of TPVB.

Thoracic epidural analgesia and TPVB represent the gold standards for both open thoracotomy and VATS. Recently, these regional anesthetic techniques, as adjuncts to anesthesia, are considered one of the critical components in a thoracic enhanced recovery after surgery (ERAS) program because of the benefit for acute postoperative pain management.^22^ However, there are possible side effects and complications, including hypotension, respiratory depression, urinary retention, nausea and vomiting, incomplete or failed block, and in rare cases, permanent neurologic injury.^4^ Recently, TEA and TPVB have been shown to provide similar pain relief following VATS, but TPVB has fewer side effect compared to TEA.^23^ In addition, a network meta-analysis on regional analgesic techniques for VATS reported that TPVB is one of the best analgesic methods, and ESP block could reduce postoperative pain within 6 hours.^24^

However, continuous TEA has been our institution’s 1st regional analgesic option for thoracic surgery. In addition, it could reduce chronic postthoracotomy pain and provide effective acute pain relief.^25^ Thus we chose continuous TEA for Comparison with the ESP block in this clinical trial.

The analgesic effect of the ESP block may be comparable to or less than that of a TPVB. Turhan et al^26^ reported that dynamic pain scores were significantly lower in TPVB compared with ESP block for 24 hours postoperatively when a single injection method was done. However, Taketa et al^27^ showed that the analgesic effect of continuous ESP block was comparable to that of TPVB for VATS using a non-inferiority test. Our study demonstrated that the postoperative NRS score between groups was generally comparable, but the TEA group had a lower NRS score than the ESP block group in the immediate postoperative period when the greatest pain intensity was expected, and the TEA showed better patient satisfaction with pain control than ESP block. In addition, total rescue morphine consumption in the ESP block group was higher than in the TEA group. Therefore, continuous ESP block may be inferior to TEA for analgesia following VATS. Our findings are consistent with the previous study that postoperative pain at rest and coughing in continuous ESP block were significantly higher than TEA 24 hours after surgery.^28^ On the contrary, Nagaraja et al^10^ have reported comparable pain scores in both TEA and ESP block groups in the early postoperative period after cardiac surgery and that an ESP block might be superior to TEA at 24 h, 36 h, and 48 h, both at rest and while coughing.

Although ESP block patients had more morphine consumption than TEA patients (6 mg versus [vs.] 3 mg), the overall incidence of adverse events in TEA patients was higher than in ESP block patients in our study. Postoperative opioid use can increase the incidence of nausea and vomiting. However, the amount of morphine consumed in both groups was relatively low because all participants took multimodal analgesia, including regional analgesic techniques, regular parenteral analgesics, and rescue opioids. In addition, the high thoracic epidural blockade could cause hypotension and related symptoms, including nausea and dizziness. Furthermore, nausea and vomiting could be related to hyperperistalsis secondary to unopposed vagal activity after TEA.^29^ Therefore, the difference in adverse events between the 2 groups is presumed to be due to these factors.

An ESP block might be a simpler and safer option to standard techniques since the key structure (the tip of the transverse process) is easily identified with ultrasound, and the needle can be inserted using the in-plane method. Additionally, the injection site is distant from the spinal cord, pleura, and major vascular structures. It may be performed at any time during the perioperative period on prone or lateral patients. Therefore, this block could provide better analgesia in patients with morbid obesity, spinal deformities, or decreased respiratory depression and could reduce the risk for patients with coagulation disorders or current anticoagulation therapy.^14,17^ Our study showed a tendency toward shorter procedural times in the ESP block group (130 s vs. 145 s). Also, none of the ESP blocks failed.

Previous literature described spreading across 5 to 9 dermatomes when the ESP block was performed at the T5 level, and 20 mL of continuous local anesthetic infusion was administered at 8 mL/h.^7,18^ The dermatomal spread of our study was an average of 4 sensory segments, which was less extensive than in previous studies. The volume of local anesthetics and different delivery protocols might affect the dermatomal range. We used only 10 mL of saline to open the interfascial plane and injected the loading dose through a multi-orifice catheter. Moreover, the background PIBI provided a maximal flow rate of 250 mL/h, much slower than the manual injection, and was started 3 hours after the injection of 20 mL of the loading dose. We presumed this prohibited an extensive spread of local anesthetics compared to previous case reports.

We used different methods to deliver local anesthetics to each group. In the ESP block group, a background PIBI with PCA was administered, whereas in the TEA group, PCA was administered continuously. The difference in the delivery method might affect the analgesic efficacy between groups. However, we expected the PIBI to result in a more extensive spread of local anesthetic and to show a better analgesic profile than continuous infusion in the ESP group. These issues have been controversial, but recently TPVB with PIBI showed a superior analgesic profile compared to continuous infusion in patients undergoing VATS.^30^

Furthermore, PIBI with TEA increased hypotension incidence, although it reduced local anesthetic consumption in patients undergoing thoracic surgery.^31^ And the patients undergoing VATS usually received 0.2% ropivacaine 5-8 ml/h as either PIBI or continuous infusion combined with a PCA pump.^30^ Thus, we programmed 0.2% ropivacaine 15ml/3h with PIBI for pain management.

### Study limitations

First, the differences in NRS between groups for the overall postoperative period were statistically significant, but we could not find them at a specific time. This result could be associated with statistical power. Thus, there is a need for further research in which sample size is added to increase statistical power. Second, 2 different analgesic procedures were performed with different positions (ESPB prone position vs. TEA lateral decubitus position). Thus this study had a weakness in the double-blinded method. However, the patients did not know which group they belonged to because it was based on their lack of prior knowledge in ESPB or TEA. Third, it was difficult to check the dermatomal sensory range, especially in the ESP block. We assessed dermatome sensitivity less than 1 h after surgery when the patient might not be fully conscious. Furthermore, the extent of the cutaneous block has some interindividual variability based on local anesthetic spread into the interfascial plane.^6^

Our results imply that ESP blocks could be an alternative to conventional techniques in patients with VATS due to their simplicity and safety, especially as a part of a multimodal analgesic strategy. Therefore, ESP blocks should be considered for routine use in patients undergoing thoracic surgery if TEA or TPVB are not considered or are contraindicated. However, further studies are necessary to confirm the efficacy of ESP blocks compared to other techniques and to establish the optimal dose and regimen.

In conclusion, continuous ESP block might be inferior to TEA; however, this technique may have tolerable analgesic efficacy and a better side effect profile than continuous epidural analgesia. Therefore, it could be an alternative to conventional regional analgesic techniques as a component of multimodal analgesia.
